# Electrocardiogram generation with a bidirectional LSTM-CNN generative adversarial network

**DOI:** 10.1038/s41598-019-42516-z

**Published:** 2019-05-01

**Authors:** Fei Zhu, Fei Ye, Yuchen Fu, Quan Liu, Bairong Shen

**Affiliations:** 1 School of Computer Science and Technology, Soochow University, Suzhou, 215006 China; 2 Provincial Key Laboratory for Computer Information Processing Technology, Soochow University, Suzhou, 215006 China; 3 School of Computer Science and Engineering, Changshu Institute of Technology, Changshu, 215500 China; 4 Institutes for Systems Genetics, West China Hospital, Sichuan University, Chengdu, 610041 China

**Keywords:** Bioinformatics, Interventional cardiology, Scientific data

## Abstract

Heart disease is a malignant threat to human health. Electrocardiogram (ECG) tests are used to help diagnose heart disease by recording the heart’s activity. However, automated medical-aided diagnosis with computers usually requires a large volume of labeled clinical data without patients' privacy to train the model, which is an empirical problem that still needs to be solved. To address this problem, we propose a generative adversarial network (GAN), which is composed of a bidirectional long short-term memory(LSTM) and convolutional neural network(CNN), referred as BiLSTM-CNN,to generate synthetic ECG data that agree with existing clinical data so that the features of patients with heart disease can be retained. The model includes a generator and a discriminator, where the generator employs the two layers of the BiLSTM networks and the discriminator is based on convolutional neural networks. The 48 ECG records from individuals of the MIT-BIH database were used to train the model. We compared the performance of our model with two other generative models, the recurrent neural network autoencoder(RNN-AE) and the recurrent neural network variational autoencoder (RNN-VAE). The results showed that the loss function of our model converged to zero the fastest. We also evaluated the loss of the discriminator of GANs with different combinations of generator and discriminator. The results indicated that BiLSTM-CNN GAN could generate ECG data with high morphological similarity to real ECG recordings.

## Introduction

Cardiovascular diseases are the leading cause of death throughout the world. Approximately 32.1% of the annual global deaths reported in 2015 were related with cardiovascular diseases^1^. Due to increases in work stress and psychological issues, the incidences of cardiovascular diseases have kept growing among young people in recent years. As an effective method, Electrocardiogram (ECG) tests, which provide a diagnostic technique for recording the electrophysiological activity of the heart over time through the chest cavity via electrodes placed on the skin^2^, have been used to help doctors diagnose heart diseases. However, as vast volumes of ECG data are generated each day and continuously over 24-hour periods^3^, it is really difficult to manually analyze these data, which calls for automatic techniques to support the efficient diagnosis of heart diseases.

Machine learning is employed frequently as an artificial intelligence technique to facilitate automated analysis. Many machine learning techniques have been applied to medical-aided diagnosis, such as support vector machines^4^, decision trees^5^, random conditional fields^6^, and recently developed deep learning methods^7^. However, most of these methods require large amounts of labeled data for training the model, which is an empirical problem that still needs to be solved. For example, large volumes of labeled ECG data are usually required as training samples for heart disease classification systems. Moreover, when machine learning approaches are applied to personalized medicine research, such as personalized heart disease research, the ECGs are often categorized based on the personal features of the patients, such as their gender and age. Thus, the problems caused by lacking of good ECG data are exacerbated before any subsequent analysis. Furthermore, maintaining the privacy of patients is always an issue that cannot be igored. However, the personal information and private clinical data obtained from patients are still likely to be illegally leaked. An optimal solution is to generate synthetic data without any private details to satisfy the requirements for research.

Hence, it is very necessary to develop a suitable method for producing practical medical samples for disease research, such as heart disease. Several previous studies have investigated the generation of ECG data. McSharry *et al*. proposed a dynamic model based on three coupled ordinary differential equations^8^, where real synthetic ECG signals can be generated by specifying heart rate or morphological parameters for the PQRST cycle. Clifford *et al*. used a nonlinear model to generate 24-hour ECG, blood pressure, and respiratory signals with realistic linear and nonlinear clinical characteristics^9^. Cao *et al.* designed an ECG system for generating conventional 12-lead signals^10^. However, most of these ECG generation methods are dependent on mathematical models to create artificial ECGs, and therefore they are not suitable for extracting patterns from existing ECG data obtained from patients in order to generate ECG data that match the distributions of real ECGs.

The generative adversarial network (GAN) proposed by Goodfellow in 2014 is a type of deep neural network that comprises a generator and a discriminator^11^. The generator produces data based on the noise data sampled from a Gaussian distribution, which is fitted to the real data distribution as accurately as possible. The inputs for the discriminator are real data and the results produced by the generator, where the aim is to determine whether the input data are real or fake. During the training process, the generator and the discriminator play a zero-sum game until they converge. GAN has been shown to be an efficient method for generating data, such as images.

In this study, we propose a novel model for automatically learning from existing data and then generating ECGs that follow the distribution of the existing data so the features of the existing data can be retained in the synthesized ECGs. Our model is based on a GAN architecture which is consisted of a generator and a discriminator. In the generator part, the inputs are noise data points sampled from a Gaussian distribution. We build up two layers of bidirectional long short-term memory (BiLSTM) networks^12^, which has the advantage of selectively retaining the history information and current information. Moreover, to prevent over-fitting, we add a dropout layer. In the discriminator part, we classify the generated ECGs using an architecture based on a convolutional neural network (CNN). The discriminator includes two pairs of convolution-pooling layers as well as a fully connected layer, a softmax layer, and an output layer from which a binary value is determined based on the calculated one-hot vector. We used the MIT-BIH arrhythmia data set^13^ for training. The results indicated that our model worked better than the other two methods, the deep recurrent neural network-autoencoder (RNN-AE)^14^ and the RNN-variational autoencoder (RNN-VAE)^15^.

## Related Work

### Generative Adversarial Network

**Figure 1:**
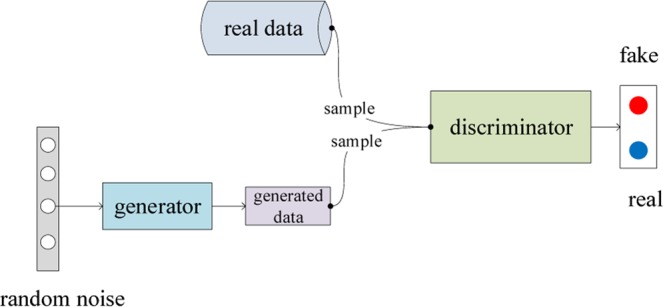
Architecture of the GAN.

The GAN is a deep generative model that differs from other generative models such as autoencoder in terms of the methods employed for generating data and is mainly comprised of a generator and a discriminator. The generator produces data based on sampled noise data points that follow a Gaussian distribution and learns from the feedback given by the discriminator. The discriminator learns the probability distribution of the real data and gives a true-or-false value to judge whether the generated data are real ones. The two sub-models comprising the generator and discriminator reach a convergence state by playing a zero-sum game. Figure 1 illustrates the architecture of GAN.

The solution obtained by GAN can be viewed as a min-max optimization process. The objective function is:

1 $$\mathop{min}\limits_{G}\,\mathop{max}\limits_{D}\,V(D,G)={E}_{x\sim {p}_{data}(x)}[\,{\rm{l}}{\rm{o}}{\rm{g}}\,D(x)]+{E}_{z\sim {p}_{z}(z)}[\,{\rm{l}}{\rm{o}}{\rm{g}}(1-D(G(z)))],$$

where *D* is the discriminator and *G* is the generator. When the distribution of the real data is equivalent to the distribution of the generated data, the output of the discriminator can be regarded as the optimal result.

GAN has been successfully applied in several areas such as natural language processing^16,17^, latent space learning^18^, morphological studies^19^, and image-to-image translation^20^.

### RNN

Recurrent neural network has been widely used to solve tasks of processing time series data^21^, speech recognition^22^, and image generation^23^. Recently, it has also been applied to ECG signal denoising and ECG classification for detecting obstructions in sleep apnea^24^. RNN typically includes an input layer, a hidden layer, and an output layer, where the hidden state at a certain time *t* is determined by the input at the current time as well as by the hidden state at a previous time:

2 $${h}_{t}=f({W}_{ih}{x}_{t}+{W}_{hh}{h}_{t-1}+{b}_{h}),$$

3 $${o}_{t}=g({W}_{ho}{h}_{t}+{b}_{o}),$$

where *f* and *g* are the activation functions, *x*_*t*_ and *o*_*t*_ are the input and output at time *t*, respectively, *h*_*t*_ is the hidden state at time *t*, *W*_{*ih,hh,ho*}_ represent the weight matrices that connect the input layer, hidden layer, and output layer, and *b*_{*h,o*}_ denote the basis of the hidden layer and output layer.

RNN is highly suitable for short-term dependent problems but is ineffective in dealing with long-term dependent problems. The long short-term memory (LSTM)^25^ and gated recurrent unit (GRU)^26^ were introduced to overcome the shortcomings of RNN, including gradient expansion or gradient disappearance during training. The LSTM is a variation of an RNN and is suitable for processing and predicting important events with long intervals and delays in time series data by using an extra architecture called the memory cell to store previously captured information. LSTM has been applied to tasks based on time series data such as anomaly detection in ECG signals^27^. However, LSTM is not part of the generative models and no studies have employed LSTM to generate ECG data yet. The GRU is also a variation of an RNN, which combines the forget gate and input gate into an update gate to control the amount of information considered from previous time flows at the current time. The reset gate of the GRU is used to control how much information from previous times is ignored. GRUs have been applied in some areas in recent years, such as speech recognition^28^.

### RNN-AE and RNN-VAE

The autoencoder and variational autoencoder (VAE) are generative models proposed before GAN. Besides used for generating data^29^, they were utilized to dimensionality reduction^30,31^.

**Figure 2:**
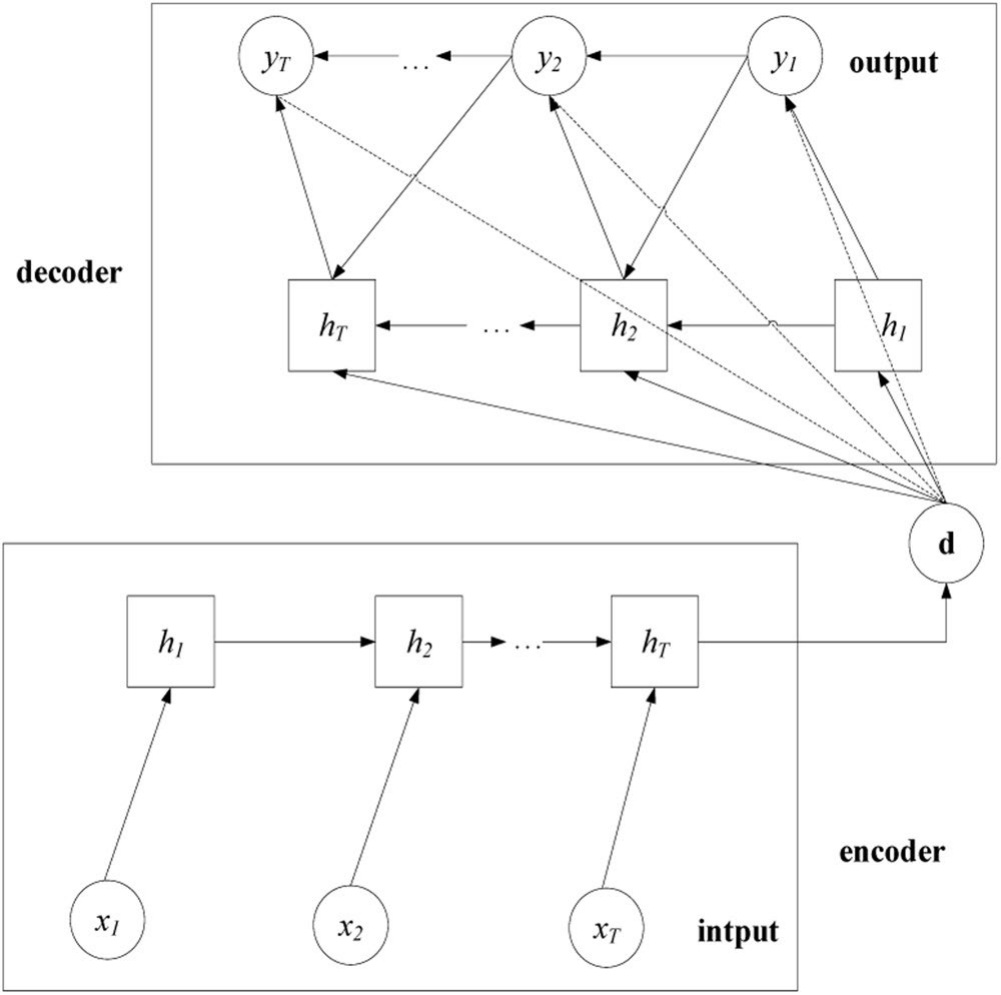
Illustration of the RNN-AE architecture.

RNN-AE is an expansion of the autoencoder model where both the encoder and decoder employ RNNs. The encoder outputs a hidden latent code **d**, which is one of the input values for the decoder. In contrast to the encoder, the output and hidden state of the decoder at the current time depend on the output at the current time and the hidden state of the decoder at the previous time as well as on the latent code **d**. The goal of RNN-AE is to make the raw data and output for the decoder as similar as possible. Figure 2 illustrates the RNN-AE architecture^14^.

VAE is a variant of autoencoder where the decoder no longer outputs a hidden vector, but instead yields two vectors comprising the mean vector and variance vector. A skill called the re-parameterization trick^32^ is used to re-parameterize the random code **z** as a deterministic code, and the hidden latent code **d** is obtained by combining the mean vector and variance vector:

4 $${\bf{d}}{\boldsymbol{=}}\mu {\boldsymbol{+}}\sigma \odot \varepsilon {\boldsymbol{,}}$$

where *μ* is the mean vector, *σ* is the variance vector, and *ε* ~ *N*(0, 1).

RNN-VAE is a variant of VAE where a single-layer RNN is used in both the encoder and decoder. This model is suitable for discrete tasks such as sequence-to-sequence learning and sentence generation.

### Generation of Time Series Data

To the best of our knowledge, there is no reported study adopting the relevant techniques of deep learning to generate or synthesize ECG signals, but there are some related works on the generation of audio and classic music signals.

Methods for generating raw audio waveforms were principally based on the training autoregressive models, such as Wavenet^33^ and SampleRNN^34^, both of them using conditional probability models, which means that at time *t* each sample is generated according to all samples at previous time steps. However, autoregressive settings tend to result in slow generation because the output audio samples have to be fed back into the model once each time, while GAN is able to avoid this disadvantage by constantly adversarial training to make the distribution of generated results and real data as approximate as possible.

Mogren *et al*. proposed a method called C-RNN-GAN^35^ and applied it on a set of classic music. In their work, tones are represented as quadruplets of frequency, length, intensity and timing. Both the generator and the discriminator use a deep LSTM layer and a fully connected layer. Inspired by their work, in our research, each point sampled from ECG is denoted by a one-dimensional vector of the time-step and leads. Donahue *et al*. applied WaveGANs^36^ from aspects of time and frequency to audio synthesis in an unsupervised background. WaveGAN uses a one-dimensional filter of length 25 and a great up-sampling factor. However, it is essential that these two operations have the same number of hyper parameters and numerical calculations. According to the above analysis, our architecture of GAN will adopt deep LSTM layers and CNNs to optimize generation of time series sequence.

## Model Design

### Overview of the Model

We propose a GAN-based model for generating ECGs. Our model comprises a generator and a discriminator. The input to the generator comprises a series of sequences where each sequence is made of 3120 noise points. The output is a generated ECG sequence with a length that is also set to 3120. The input to the discriminator is the generated result and the real ECG data, and the output is *D*(*x*) ∈ {0, 1}. In the training process, *G* is initially fixed and we train *D* to maximize the probability of assigning the correct label to both the realistic points and generated points. We then train *G* to minimize log(1 − *D*(*G*(*z*))). The objective function is described by Eq. 5:

5 $$\mathop{{\rm{\min }}}\limits_{{G}_{\theta }}\,\mathop{{\rm{\max }}}\limits_{{D}_{\varphi }}\,{L}_{\theta ;\varphi }=\frac{1}{N}\sum _{i=1}^{N}[\,\mathrm{log}\,{D}_{\varphi }({x}_{i})+(\mathrm{log}(1-{D}_{\varphi }({G}_{\theta }({z}_{i}))))],$$

where *N* is the number of points, which is 3120 points for each sequence in our study, and *θ* and *ϕ* represent the set of parameters.

As CNN does not have recurrent connections like forgetting units as in LSTM or GRU, the training process of the models with CNN-based discriminator is often faster, especially in the case of long sequence data modeling. CNN has achieved excellent performance in sequence classification such as the text or voice sorting^37^. Many successful deep learning methods applied to ECG classification and feature extraction are based on CNN or its variants. Therefore, the CNN discriminator is nicely suitable to the ECG sequences data modeling.

### Design of the Generator

A series of noise data points that follow a Gaussian distribution are fed into the generator as a fixed length sequence. We assume that each noise point can be represented as a *d*-dimensional one-hot vector and the length of the sequence is *T*. Thus, the size of the input matrix is *T* × *d*.

**Figure 3:**
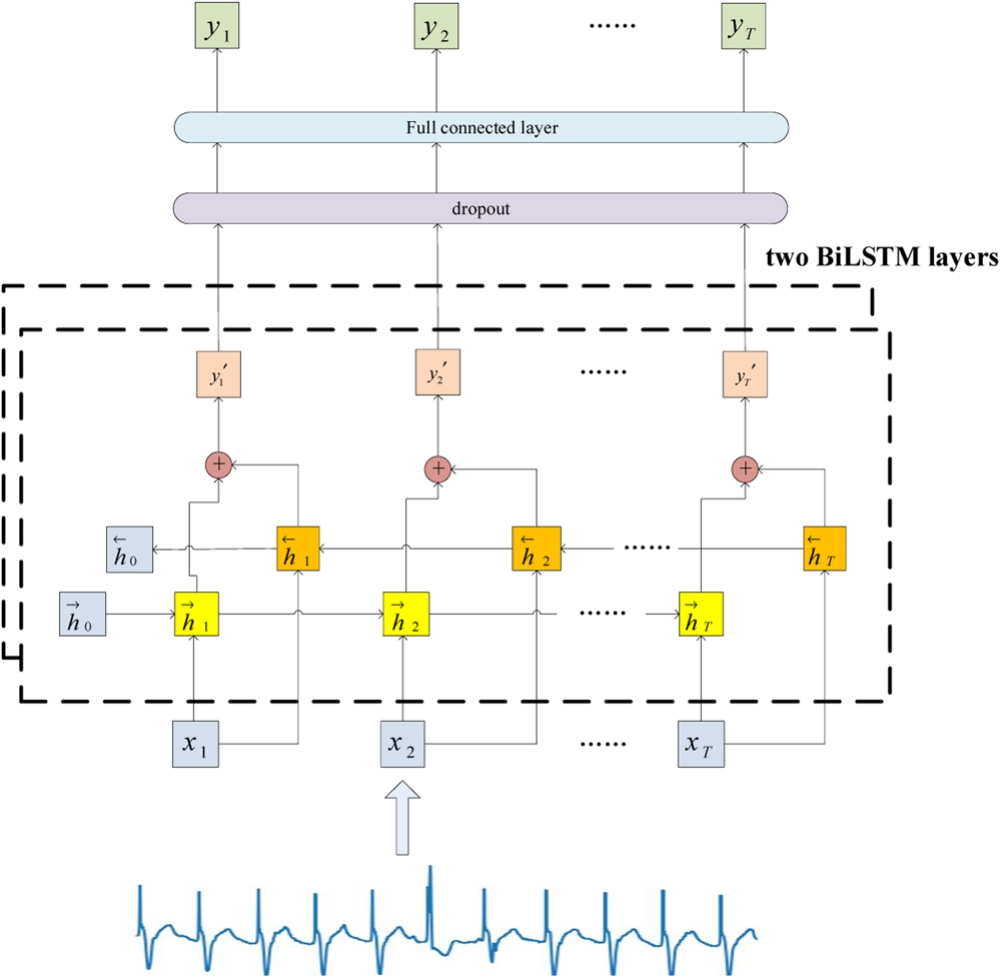
Architecture of the generator.

The generator comprises two BiLSTM layers, each having 100 cells. A dropout layer is combined with a fully connected layer. The architecture of the generator is shown in Fig. 3.

The current hidden state depends on two hidden states, one from forward LSTM and the other from backward LSTM. Eqs 6 and 7 are used to calculate the hidden states from two parallel directions and Eq. 9 calculates the output of the first BiLSTM layer at time *t*:

6 $$\overrightarrow{{h}_{t}^{1}}=\,\tanh ({W}_{i\overrightarrow{h}}^{1}{x}_{t}+{W}_{\overrightarrow{h}\overrightarrow{h}}^{1}{h}_{t-1}^{\overrightarrow{1}}+{b}_{\overrightarrow{h}}^{1}),$$

7 $$\overleftarrow{{h}_{t}^{1}}=\,\tanh ({W}_{i\overleftarrow{h}}^{1}{x}_{t}+{W}_{\overleftarrow{h}\overleftarrow{h}}^{1}\,{h}_{t+1}^{\overleftarrow{1}}+{b}_{\overleftarrow{h}}^{1}),$$

8 $${y}_{t}^{1}=\,\tanh ({W}_{\overrightarrow{h}o}^{1}\overrightarrow{{h}_{t}^{1}}+{W}_{\overleftarrow{h}o}^{1}\overleftarrow{{h}_{t}^{1}}+{b}_{o}^{1}),$$

where the output depends on ${\overrightarrow{h}}_{t}$ and ${\overleftarrow{h}}_{t}$, and *h*_0_ is initialized as a zero vector.

Similarly, we obtain the output at time *t* from the second BiLSTM layer:

9 $${y}_{t}=\,\tanh ({W}_{\overrightarrow{h}o}^{2}\,\overrightarrow{{h}_{t}^{2}}+{W}_{\overleftarrow{h}o}^{2}\,\overleftarrow{{h}_{t}^{2}}+{b}_{o}^{2}).$$

To prevent slow gradient descent due to parameter inflation in the generator, we add a dropout layer and set the probability to 0.5^38^. The output layer is a two-dimensional vector where the first element represents the time step and the second element denotes the lead.

### Design of the Discriminator

**Figure 4:**
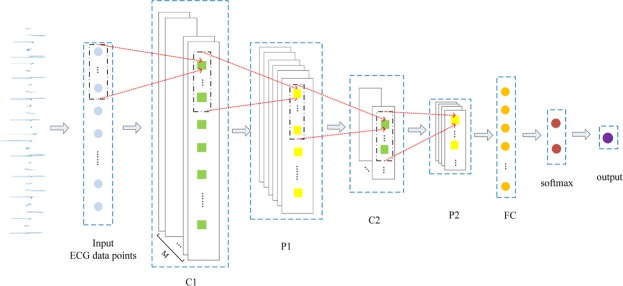
Structure of the CNN in the discriminator.

The architecture of discriminator is illustrated in Fig. 4. The pair of red dashed lines on the left denote a type of mapping indicating the position where a filter is moved, and those on the right show the value obtained by using the convolution operation or the pooling operation.

The sequence comprising ECG data points can be regarded as a time series sequence (a normal image requires both a vertical convolution and a horizontal convolution) rather than an image, so only one-dimensional (1-D) convolution need to be involved. We assume that an input sequence *x*_1_, *x*_2_, … *x*_*T*_ comprises *T* points, where each is represented by a *d*-dimensional vector. We set the size of filter to h*1, the size of the stride to k*1 (*k* ≪ *h*), and the number of the filters to *M*. Therefore, the output size from the first convolutional layer is *M* * [(*T* − *h*)/*k* + 1] * 1. The window for the filter is:

10 $${x}_{l:r}={x}_{l}\oplus {x}_{l+1}\oplus {x}_{l+2}\oplus \ldots \oplus {x}_{r}.$$

The values of *l* and *r* are determined by:

11 $$l=k\ast i+1-k\,\,l\in [1,\,T-h+1],$$

12 $$r=k\ast i-k+h\,\,r\in [h,T],$$

where 1 ≤ *k* * *i* + 1 ≤ *T* − *h* + 1 and *h* ≤ *k* * *i* − *k* + *h* ≤ *T* (*i* ∈ [1, (*T* − *h*)/*k* + 1]).

The returned convolutional sequence *c* = [*c*_1_, *c*_2_, … *c*_*i*_, …] with each *c*_*i*_ is calculated as

13 $${c}_{i}=f(w\ast {x}_{l:r}+b),$$

where $w\in {{\mathbb{R}}}^{h\times d}$ a shared weight matrix, and *f* represents a nonlinear activation function.

The successor layer is the max pooling layer with a window size of a*1 and stride size of b*1. Each output from pooling *p*_*j*_ for the returned pooling result sequence *p* = [*p*_1_, *p*_2_, … *p*_*j*_ …] is:

14 $${p}_{j}=\,{\rm{\max }}({c}_{bj+1-b},{c}_{bj+2-b},\,\ldots \,{c}_{bj+a-b}).$$

After conducting double pairs of operations for convolution and pooling, we add a fully connected layer that connects to a softmax layer, where the output is a one-hot vector. The two elements in the vector represent the probability that the input is true or false. The function of the softmax layer is:

15 $$\sigma {(z)}_{j}=\frac{{e}^{{z}_{j}}}{{\sum }_{k=1}^{2}{e}^{{z}_{k}}}(j=1,\,2).$$

In Table 1, C1 layer is a convolutional layer, with the size of each filter 120*1, the number of filters is 10 and the size of stride is 5*1. The output size of C1 is calculated by:

16 $$\frac{(W,H)-F+2P}{S}+1,$$

where (*W*, *H*) represents the input volume size (1*3120*1), *F* and *S* denote the size of kernel filters and length of stride respectively, and *P* is the amount of zero padding and it is set to 0. Thus, the output size of C1 is 10*601*1.

**Table 1:** Parameters for each layer of the discriminator.

Layer	Feature Maps	Filter	Stride	Input Size	Output Size
Input	—	—	—	1*3120*1	—
C1	10	120*1	5*1	1*3120*1	10*601*1
P1	10	46*1	3*1	10*601*1	10*186*1
C2	5	36*1	3*1	10*186*1	5*51*1
P2	5	24*1	3*1	5*51*1	5*10*1
FC	5	—	—	5*10*1	25
Softmax	—	—	—	25	25
Output	—	—	—	25	1

In Table 1, the P1 layer is a pooling layer where the size of each window is 46*1 and size of stride is 3*1. The output size of P1 is computed by:

17 $$\frac{(W,H)-F}{S}+1,$$

where (*W*, *H*) represents the input volume size (10*601*1), *F* and *S* denote the size of each window and the length of stride respectively. Thus, calculated by Eq. 17, the output size of P1 is 10*186*1.

The computational principle of parameters of convolutional layer C2 and pooling layer P2 is the same as that of the previous layers. It needs to be emphasized that the amount of kernels filters of C2 is set to 5 factitiously. With pairs of convolution-pooling operations, we get the output size as 5*10*1. A fully connected layer which contains 25 neurons connects with P2. The last layer is the softmax-output layer, which outputs the judgement of the discriminator.

## Experiments and Analyses

### The Computing Platform

In the experiment, we used a computer with an Intel i7-7820X (8 cores) CUP, 16 GB primary memory, and a GeForce GTX 1080 Ti graphics processing unit (GPU). The operating system is Ubuntu 16.04LTS. We implemented the model by using Python 2.7, with the package of PyTorch and NumPy. Compared to the static platform, the established neural network in PyTorch is dynamic. The result of the experiment is then displayed by Visdom, which is a visual tool that supports PyTorch and NumPy.

### Representation of ECG Data

We used the MIT-BIH arrhythmia data set provided by the Massachusetts Institute of Technology for studying arrhythmia in our experiments. We downloaded 48 individual records for training. Each record comprised three files, i.e., the header file, data file, and annotation file. Each data file contained about 30 minutes of ECG data. In each record, a single ECG data point comprised two types of lead values; in this work, we only selected one lead signal for training:

18 $${x}_{t}={[{x}_{t}^{\alpha },{x}_{t}^{\beta }]}^{T},$$

19 $$v({x}_{t})={x}_{t}^{\alpha }/200,$$

where *x*_*t*_ represents the ECG points at time step *t* sampled at 360 Hz, ${x}_{t}^{\alpha }$ is the first sampling signal value, and ${x}_{t}^{\beta }$ is the second one. Both were divided by 200 to calculate the corresponding lead value. The number of ECG data points in each record was calculated by multiplying the sampling frequency (360 Hz) and duration of each record for about 650,000 ECG data points. Therefore, we used 31.2 million points in total.

### Training Results

First, we compared the GAN with RNN-AE and RNN-VAE. All of the models were trained for 500 epochs using a sequence of 3120 points, a mini-batch size of 100, and a learning rate of 10^−5^. The loss of the GAN was calculated with Eq. 5 and the loss of RNN-AE was calculated as:

20 $$\mathop{{\rm{\max }}}\limits_{\theta }=\frac{1}{N}\sum _{i=1}^{N}\mathrm{log}\,{p}_{\theta }({y}_{i}|{x}_{i}),$$

where *θ* is the set of parameters, *N* is the length of the ECG sequence, *x*_*i*_ is the i^th^ point in the sequence, which is the input of for the encoder, and *y*_*i*_ is the *i*^th^ point in the sequence, which is the output from the decoder.

The loss of RNN-VAE was calculated as:

21 $$\sum _{i=1}^{N}L(\theta ,\,\varphi :\,{x}_{i})=\sum _{i=1}^{N}-KL({q}_{\varphi }(\overrightarrow{z}|{x}_{i}))\Vert {p}_{\theta }(\overrightarrow{z})+{E}_{{q}_{\varphi }(\overrightarrow{z}|{x}_{i})}[\,\mathrm{log}\,{p}_{\theta }({x}_{i}|\overrightarrow{z})],$$

where ${p}_{\theta }(\overrightarrow{z})$ is usually a standard prior *N ~ *(0, 1), ${q}_{\varphi }(\overrightarrow{z}|x)$ is the encoder, ${p}_{\theta }(x|\overrightarrow{z})$ is the decoder, and *θ* and *ϕ* are the sets of parameters for the decoder and encoder, respectively.

**Figure 5:**
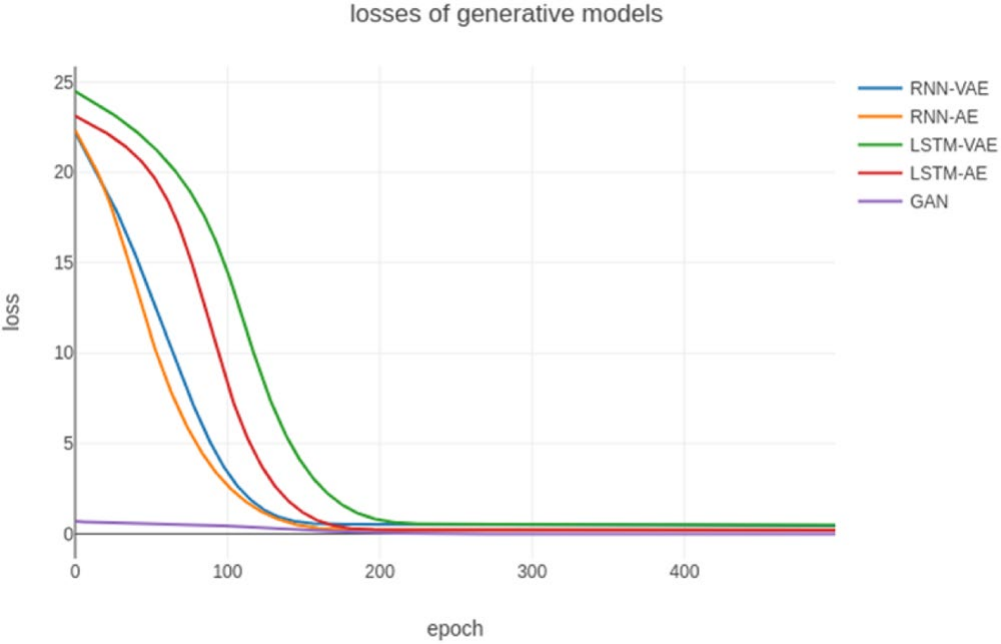
Losses of five generative models.

We extended the RNN-AE to LSTM-AE, RNN-VAE to LSTM-VAE, and then compared the changes in the loss values of our model with these four different generative models. Figure 5 shows the training results, where the loss of our GAN model was the minimum in the initial epoch, whereas all of the losses of the other models were more than 20. After 200 epochs of training, our GAN model converged to zero while other models only started to converge. At each stage, the value of the loss function of the GAN was always much smaller than the losses of the other models obviously.

**Figure 6:**
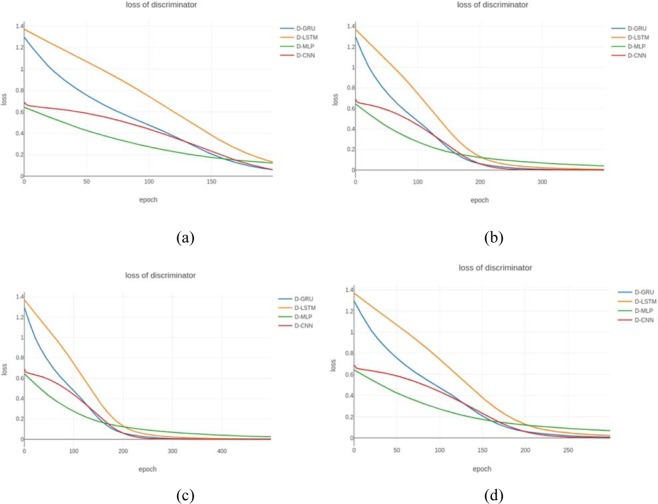
Loss of each type of discriminator. The four lines represent the discriminators based mainly on the structure with the CNN (red line), MLP (green line), LSTM (orange line), and GRU (blue line). (**a–d**) Represent the results after 200, 300, 400, and 500 epochs of training.

We then compared the results obtained by the GAN models with those using a CNN, MLP (Multi-Layer Perceptron), LSTM, and GRU as discriminators, which we denoted as BiLSTM-CNN, BiLSTM-GRU, BiLSTM-LSTM, and BiLSTM-MLP, respectively. Each model was trained for 500 epochs with a batch size of 100, where the length of the sequence comprised a series of ECG 3120 points and the learning rate was 1 × 10^−5^. Figure 6 shows the losses calculated of the four GAN discriminators using Eq. 5.

Figure 6 shows that the loss with the MLP discriminator was minimal in the initial epoch and largest after training for 200 epochs. The loss with the discriminator in our model was slightly larger than that with the MLP discriminator at the beginning, but it was obviously less than those of the LSTM and GRU discriminators. Eventually, the loss converged rapidly to zero with our model and it performed the best of the four models.

### ECG Generation

**Figure 7:**
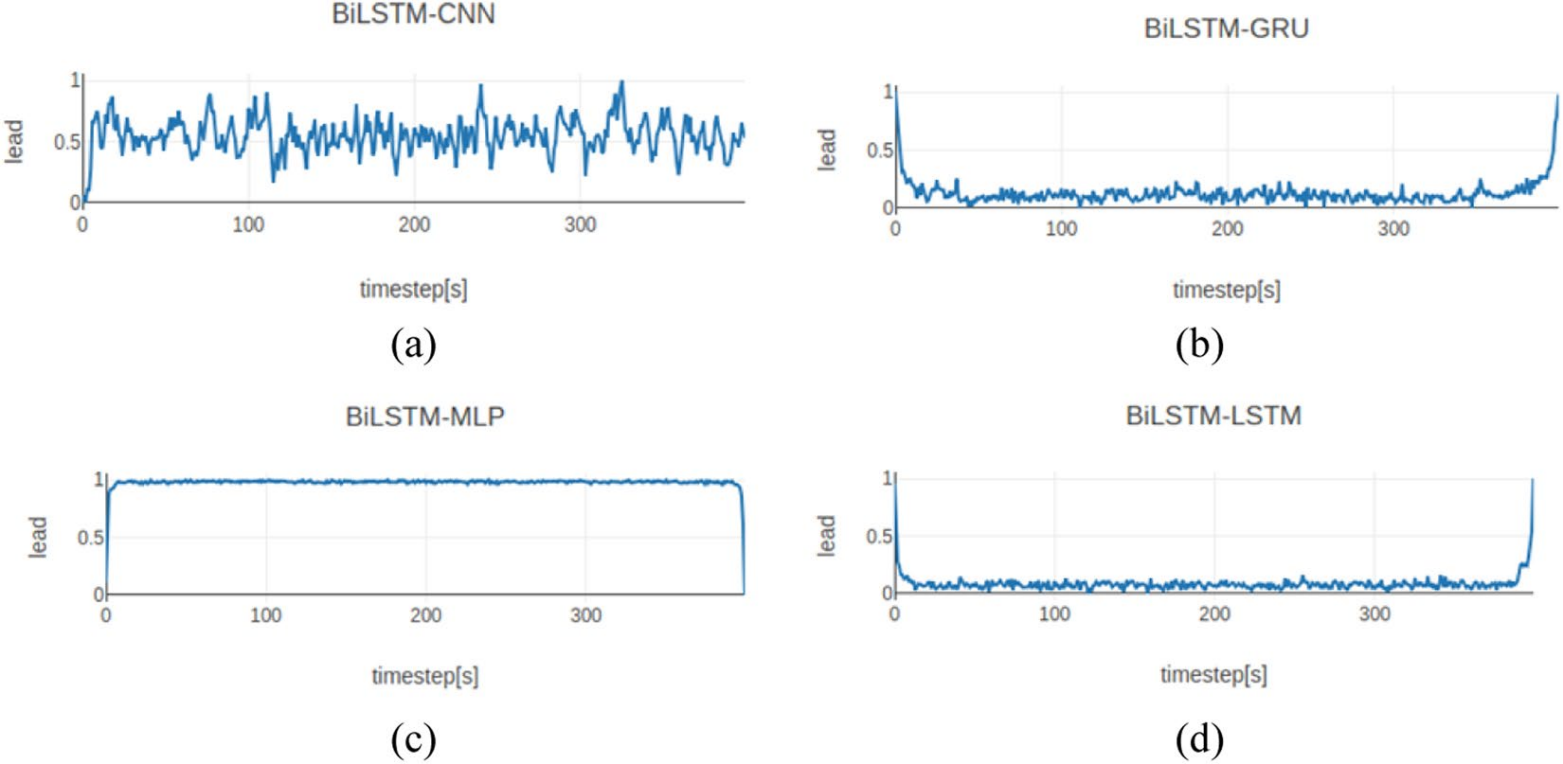
Results generated using different discriminator structures. (**a–d**) Represent the results obtained when the discriminator used the CNN, GRU, MLP, and LSTM respectively.

Finally, we used the models obtained after training to generate ECGs by employing the GAN with the CNN, MLP, LSTM, and GRU as discriminators. The dim for the noise data points was set to 5 and the length of the generated ECGs was 400. Figure 7 shows the ECGs generated with different GANs.

Figure 7 shows that the ECGs generated by our proposed model were better in terms of their morphology. We found that regardless of the number of time steps, the ECG curves generated using the other three models were warped up at the beginning and end stages, whereas the ECGs generated with our proposed model were not affected by this problem.

We then evaluated the ECGs generated by four trained models according to three criteria. The distortion quantifies the difference between the original signal and the reconstructed signal. We evaluated the difference between the real data and the generated points with the percent root mean square difference (PRD)^39^, which is the most widely used distortion measurement method.

The generated points were first normalized by:

22 $${x}_{[n]}=\frac{{x}_{[n]}-{x}_{{\rm{\max }}}}{{x}_{{\rm{\max }}}-{x}_{{\rm{\min }}}}.$$

The PRD was calculated as:

23 $$PRD=\sqrt{\frac{{\sum }_{n=1}^{N}{({x}_{[n]}-\widehat{{x}_{[n]}})}^{2}}{{\sum }_{n=1}^{N}{({x}_{[n]})}^{2}}\times 100,}$$

where *x*_[*n*]_ is the *n*^th^ real point, $\widehat{{x}_{[n]}}$ is the *n*^th^ generated point, and *N* is the length of the generated sequence.

The root mean square error (RMSE)^39^ reflects the stability between the original data and generated data, and it was calculated as:

24 $$RMSE=\sqrt{\frac{1}{N}{\sum }_{n=1}^{N}{({x}_{[n]}-\widehat{{x}_{[n]}})}^{2}.}$$

The Fréchet distance (FD)^40^ is a measure of similarity between curves that takes into consideration the location and ordering of points along the curves, especially in the case of time series data. A lower FD usually stands for higher quality and diversity of generated results.

Let *P* be the order of points along a segment of realistic ECG curve, and *Q* be the order of points along a segment of a generated ECG curve: $\sigma (P)=({u}_{1},\,{u}_{2},\,\mathrm{...}\,{u}_{p})$, $\sigma (Q)=({\nu }_{1},\,{\nu }_{2},\,\mathrm{...}\,{\nu }_{q})$. Then we can get a sequence which consists of couple of points: $\{({u}_{{a}_{1}},{v}_{{b}_{1}}),\,\mathrm{...}({u}_{{a}_{m}},{v}_{{b}_{m}})\}$. The length $||d||$ of this sequence is computed by:

25 $$||d||=\mathop{{\rm{\max }}}\limits_{i=1,\mathrm{...}m}\,d({u}_{{a}_{i}},{v}_{{b}_{i}}),$$

where *d* represents the Euclidean distance. Essentially, we have ${a}_{i+1}={a}_{i}$ or ${a}_{i+1}={a}_{i}+1$ and ${b}_{i+1}={b}_{i}$ as prerequisites.

Finally, the discrete Fréchet distance is calculated as:

26 $$FD(P,Q)=\,{\rm{\min }}\,\{||d||\}$$

Table 2 shows that our model has the smallest metric values about PRD, RMSE and FD compared with other generative models.

**Table 2:** Results of evaluate metrics for different generative models.

Method	PRD	RMSE	FD
BILSTM-CNN GAN	**66.408**	**0.276**	**0.756**
RNN-AE GAN	121.877	0.506	0.969
LSTM-AE GAN	148.650	0.618	0.996
RNN-VAE GAN	146.566	0.609	0.982
LSTM-VAE GAN	145.978	0.607	0.975

We can see that the FD metric values of other four generative models fluctuate around 0.950. The RMSE and PRD of these models are much smaller than that of the BiLSTM-CNN GAN. This indicates that except for RNN-AE, the corresponding PRD and RMSE of LSTM-AE, RNN-VAE, LSTM-VAE are fluctuating between 145.000 to 149.000, 0.600 to 0.620 respectively because of their similar architectures. Based on the results shown in Table 2, we can conclude that our model is the best in generating ECGs compared with different variants of the autocoder. Table 3 shows that our proposed model performed the best in terms of the RMSE, PRD and FD assessment compared with different GANs.

**Table 3:** Results of evaluate metrics for GANs with different discriminators.

Method	PRD	RMSE	FD
BiLSTM-CNN GAN	**51.799**	**0.215**	**0.803**
BiLSTM-GRU	74.047	0.308	0.853
BiLSTM-LSTM	84.795	0.352	0.901
BiLSTM-MLP	147.732	0.614	0.989

Table 3 demonstrated that the ECGs obtained using our model were very similar to the standard ECGs in terms of their morphology. In addition, the LSTM and GRU are both variations of RNN, so their RMSE and PRD values were very similar.

**Figure 8:**
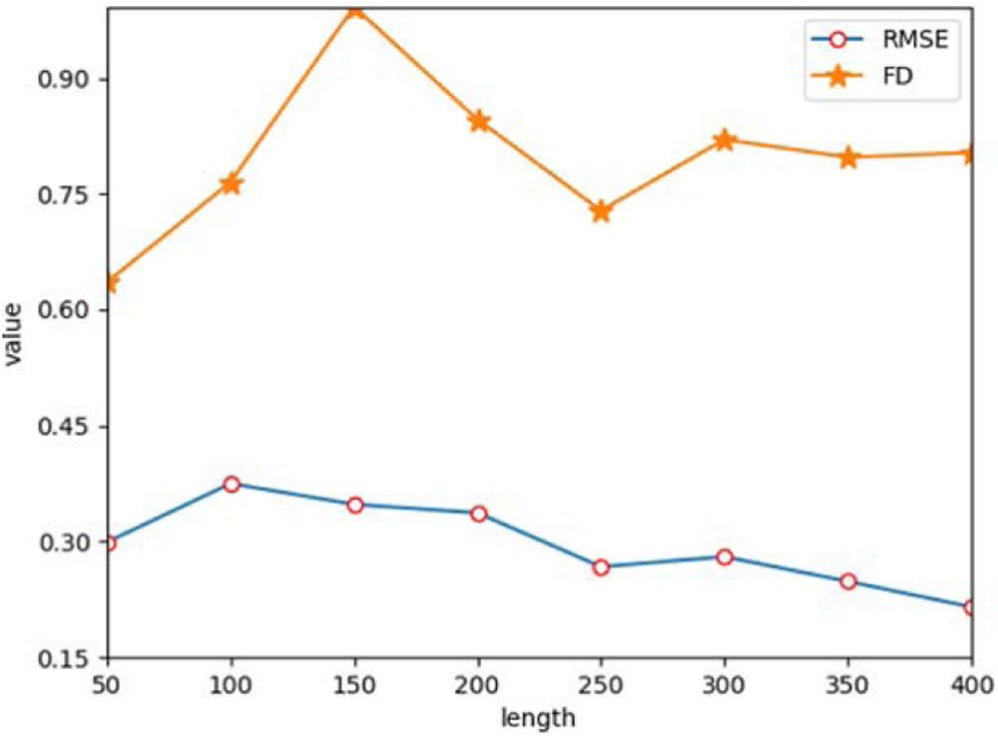
Results of RMSE and FD by different specified lengths.

From the results listed in Tables 2 and 3, we can see that both of RMSE and FD values are between 0 and 1. Under the BiLSTM-CNN GAN, we separately set the length of the generated sequences and obtain the corresponding evaluation values. It is well known that under normal circumstances, the average heart rate is 60 to 100 in a second. Therefore, the normal cardiac cycle time is between 0.6 s to 1 s. Based on the sampling rate of the MIT-BIH, the calculated length of a generated ECG cycle is between 210 and 360. Figure 8 shows the results of RMSE and FD by different specified lengths from 50–400. From Fig. 8, we can conclude that the quality of generation is optimal when the generated length is 250 (RMSE: **0.257**, FD: **0.728**).

## Conclusion

To address the lack of effective ECG data for heart disease research, we developed a novel deep learning model that can generate ECGs from clinical data without losing the features of the existing data. Our model is based on the GAN, where the BiLSTM is used as the generator and the CNN is used as the discriminator. After training with ECGs, our model can create synthetic ECGs that match the data distributions in the original ECG data. Our model performed better than other two deep learning models in both the training and evaluation stages, and it was advantageous compared with other three generative models at producing ECGs. The ECGs synthesized using our model were morphologically similar to the real ECGs.
